# Comparison of registered and published intervention fidelity assessment in cluster randomised trials of public health interventions in low- and middle-income countries: systematic review

**DOI:** 10.1186/s13063-018-2796-z

**Published:** 2018-07-31

**Authors:** Myriam Cielo Pérez, Nanor Minoyan, Valéry Ridde, Marie-Pierre Sylvestre, Mira Johri

**Affiliations:** 1 Centre de Recherche du Centre Hospitalier de l’Université de Montréal (CRCHUM), 900, rue Saint-Denis, Pavillon R, Tour Saint-Antoine Porte S03.414, Montréal, Québec H2X 0A9 Canada; 2 Département de médicine sociale et préventive, École de santé publique (ESPUM), Université de Montréal, 7101, avenue du Parc, 3e étage, Montréal, Québec H3N 1X9 Canada; 3 Institut de Recherche en Santé Publique Université de Montréal (IRSPUM), Pavillon 7101 Avenue du Parc, P.O. Box 6128, Centre-ville Station, Montréal, Québec H3C 3J7 Canada; 4 Institut de Recherche Pour le Développement (IRD), Le Sextant 44, bd de Dunkerque, CS 90009 13572, Cedex 02 Marseille, France; 5 Département de gestion, d’évaluation, et de politique de santé, École de santé publique, Université de Montréal, 7101, avenue du Parc, 3e étage, Montréal, Québec H3N 1X9 Canada

**Keywords:** Cluster randomised trials, Intervention fidelity, Public health interventions, Process evaluation, Developing countries, Systematic review

## Abstract

**Background:**

Cluster randomised trials (CRTs) are a key instrument to evaluate public health interventions. Fidelity assessment examines study processes to gauge whether an intervention was delivered as initially planned. Evaluation of implementation fidelity (IF) is required to establish whether the measured effects of a trial are due to the intervention itself and may be particularly important for CRTs of complex interventions conducted in low- and middle-income countries (LMICs). However, current CRT reporting guidelines offer no guidance on IF assessment. The objective of this review was to study current practices concerning the assessment of IF in CRTs of public health interventions in LMICs.

**Methods:**

CRTs of public health interventions in LMICs that planned or reported IF assessment in either the trial protocol or the main trial report were included. The MEDLINE/PubMed, CINAHL and EMBASE databases were queried from January 2012 to May 2016. To ensure availability of a study protocol, CRTs reporting a registration number in the abstract were included. Relevant data were extracted from each study protocol and trial report by two researchers using a predefined screening sheet. Risk of bias for individual studies was assessed.

**Results:**

We identified 90 CRTs of public health interventions in LMICs with a study protocol in a publicly available trial registry published from January 2012 to May 2016. Among these 90 studies, 25 (28%) did not plan or report assessing IF; the remaining 65 studies (72%) addressed at least one IF dimension. IF assessment was planned in 40% (36/90) of trial protocols and reported in 71.1% (64/90) of trial reports. The proportion of overall agreement between the trial protocol and trial report concerning occurrence of IF assessment was 66.7% (60/90). Most studies had low to moderate risk of bias.

**Conclusions:**

IF assessment is not currently a systematic practice in CRTs of public health interventions carried out in LMICs. In the absence of IF assessment, it may be difficult to determine if CRT results are due to the intervention design, to its implementation, or to unknown or external factors that may influence results. CRT reporting guidelines should promote IF assessment.

**Trial Registration:**

Protocol published and available at: 10.1186/s13643-016-0351-0

**Electronic supplementary material:**

The online version of this article (10.1186/s13063-018-2796-z) contains supplementary material, which is available to authorized users.

## Background

Cluster randomised trials (CRTs) have become a key instrument to evaluate public health interventions [1–7], particularly in low- and middle-income countries (LMICs) [3, 8]. Randomised controlled trials (RCTs) are widely considered to provide the highest quality of evidence on the effectiveness of health interventions [9–12], and CRTs are a form of randomised trial in which clusters of individuals (such as families, villages, hospital services or schools), rather than independent individuals, are randomly allocated to intervention or control groups [2]. Increasingly, public health researchers recognise the importance of developing health interventions that target populations rather than individuals and can address the wide range of social and environmental factors influencing health [13, 14]. CRTs offer an appropriate design to assess such public health interventions as well as to measure the overall effect of an intervention at the population level [3, 5, 8, 13, 15], heterogeneity of impact among population subgroups and equity [16, 17].

### Intervention fidelity in CRTs of public health interventions

Recent methodologically oriented systematic reviews have found evidence of improvements in the design and analysis of CRTs, while also noting deficiencies in trial implementation [18, 19]. Previous systematic reviews have emphasised the potential importance of process evaluation to mitigate these methodological problems, which can affect the internal and external validity of trial results [3, 18, 20–22].

‘Intervention fidelity’ refers to the degree to which an intervention is delivered as initially planned [23]. Fidelity assessment is the practice of evaluating intervention fidelity. Considered an aspect of process evaluation, fidelity assessment aims to understand and measure to what extent the intervention is being implemented as intended and identify specific reasons for the success or failure of the intervention [9, 23, 24]. The benefits of evaluating intervention fidelity include increased confidence in scientific findings, increased power to control for confounding factors and increased ability to evaluate the performance of theory-based interventions [25]. Fidelity assessment can improve internal and external validity of CRTs [18] by providing evidence that trial results are due to interventions themselves rather than to external factors and facilitating generalisation of results to contexts that may differ substantially from the original trial setting [9, 23]. Fidelity assessment may be particularly important for public health interventions, as these interventions tend to be complex and constituted by multiple components [10, 26] that act independently or interdependently [27], allowing greater potential for variation during implementation [23].

### Framework for the evaluation of implementation fidelity

Table 1 outlines the conceptual framework used in this review, based on the work of Carroll et al. [23] and refined by Hasson [28]. We selected this framework to guide the review because it provides a comprehensive synthesis of previous work on intervention fidelity and has been widely influential.

**Table 1:** Conceptual framework for intervention fidelity used in this review

Fidelity components	
Content	Defined as an attempt to establish the ‘active ingredients’ of the intervention
Coverage	Refers to the degree to which all persons who met study inclusion criteria received the intervention
Frequency	Refers to whether the intervention was delivered with the regularity or frequency planned by its designers
Duration	Establishes whether the intervention was delivered with the duration planned by its designers
Moderating factors	
Comprehensiveness of intervention description	Factors such as the degree of intervention complexity and whether the intervention description is complete or incomplete, vague or clear, may influence the degree of intervention fidelity
Strategies to facilitate implementation	Several support strategies may be used to optimise and to standardise intervention fidelity
Quality of delivery	Concerns whether an intervention is delivered in a way that increases the likelihood of achieving the desired health outcomes
Participant responsiveness	Refers to whether researchers established strategies to increase acceptance by and acceptability to those receiving an intervention
Recruitment^a^	Refers to procedures used to attract potential programme participants
Context^a^	Refers to surrounding social systems, such as structures and cultures of organisations and groups, and historical and concurrent activities and events, that may influence study activities

Adapted from Carroll et al. [23]

^a^These components were added by Hasson [28]

### Fidelity assessment in CRT reporting guidelines

The Consolidated Standards of Reporting Trials (CONSORT) group was created to provide guidance to improve the quality and transparency of RCT reporting [29], including CRTs [2, 30]. The CONSORT statement recognises that the trial protocol for a given study may not have been followed fully by some trial participants for a wide variety of reasons, including failure to receive the entire intervention as planned [29]. Cases of protocol non-adherence may influence interpretation and credibility of the results and thus the validity of the conclusions [18, 25, 31, 32]. To preserve the ability to make strong inferences about intervention effects, CONSORT recommends that all participants randomised be retained in the analysis and analysed according to their original assigned groups, an approach known as ‘intention-to-treat’ (ITT) analysis.

Reasons for protocol non-adherence in individually randomised RCTs may differ from those in CRTs. In a clinical trial setting, non-adherence depends largely on the actions of the trial participant and the treatment provider, which may in turn be related to issues such as treatment side effects and safety. In CRTs of public health interventions, protocol non-adherence may occur because complex interventions that include multiple components are delivered with poor fidelity. However, both for individually randomised parallel group trials [29] and CRTs [2, 30], current CONSORT guidelines offer no advice on methods to assess protocol non-adherence.

### Rationale for undertaking this review

As earlier methodologically oriented systematic reviews have demonstrated, CRTs of complex public health interventions may be particularly at risk of experiencing protocol deviations and non-adherence, and these may compromise the validity of their findings [18, 19]. Although process evaluation techniques, such as evaluation of intervention fidelity, can help to identify these problems and mitigate their negative effects, current reporting guidelines for CRTs offer no specific guidance on intervention fidelity assessment. Wide divergence in current practices is therefore likely. We undertook a methodologically oriented systematic review of current practices related to assessment of intervention fidelity within CRTs of public health interventions in LMICs, so as to inform best practices for CRTs. To our knowledge, no other systematic review addresses this question. Although CRTs have been widely implemented to evaluate public health interventions in both high-income countries and LMIC contexts, interventions, approaches and outcomes may differ substantially between settings. Given the limitations of the resources, these challenges may be even more important in CRTs of public health interventions in LMICs. We therefore limit our focus to LMICs.

### Objective

We conducted a methodologically oriented systematic review to address the following research questions:

1. What proportion of recent CRTs of public health interventions in LMICs planned to assess intervention fidelity (IF)?
2. What proportion of recent CRTs of public health interventions in LMICs reported assessing IF?
3. Which fidelity components were examined and which data collection methods were employed to assess each component?
4. Is there evidence of divergent practices between planned and reported studies?

## Methods

We report results according to the Preferred Reporting Items for Systematic Reviews and Meta-Analyses (PRISMA) guidelines [33]; the PRISMA checklist is available in an online supplement (Additional file 1). For a detailed description of the methods, see the published protocol [34] (doi:10.1186/s13643-016-0351-0). Some technical modifications were made while implementing the review. We report the date for each change in italics, along with the rationale.

### Study eligibility

#### Study designs

We included all CRTs. For the purposes of this review, a CRT is defined as a trial in which “*intact social units or clusters of individuals, rather than independent individuals, are randomly allocated to intervention groups*” [30]. CRTs may include trials employing parallel group, stepped wedge, factorial, adaptive or pragmatic designs, among others.

#### Participants

Study participants were humans living in LMICs according to the World Bank country classifications [35].

#### Interventions

This review focuses on ‘public health interventions’. Following Rychetnik et al. [36, 37], we define a public health intervention as a disease prevention or health promotion intervention applied to many, most or all members in a community, which aims to deliver a net benefit to the community or population, as well as benefits to individuals. Assessment of intervention fidelity may be especially important for public health interventions, which are inherently “*complex, programmatic, and context dependent*” [36]. In order to operationalise this definition and guide study inclusion decisions, we used the Minnesota Department of Health ‘Intervention Wheel’ [38].

#### Comparators

Comparators were defined as per the original CRT.

#### Outcomes

We included CRTs that assessed IF in the trial protocol, the main trial report or both. We also included CRTs reporting assessment of IF in a complementary document such as a published article, an online appendix to the main paper, or a grey literature report, in lieu of reporting assessment of IF in the main trial report only. We considered studies as evaluating IF if they proposed or implemented methods to assess at least one of the four key fidelity components, namely (1) content, (2) coverage, (3) frequency and (4) duration.

### Report characteristics

#### Availability of the study protocol

To ensure availability of a study protocol, we included CRTs reporting a registration number in the abstract for any registry meeting WHO Trial Registration Data Set criteria [39]. The Trial Registration Data Set was used in this review to evaluate planned assessment of intervention fidelity, either alone or in conjunction with a published study protocol.

#### Exclusion criteria

We excluded studies that (1) were not cluster randomised trials, (2) did not plan or report assessment of IF, (3) were not public health interventions, (4) were conducted in high-income countries [35], (5) were published before 2012, (6) did not have a publicly available protocol or (7) for which only the protocol but not the main trial report has been published. Manuscripts were also excluded if they were (8) pilot studies, (9) secondary reports of a main study for which the relevant findings were published prior to 2012, date of the last update to the CONSORT Statement to improve reporting of CRTs, (10) studies published in a language other than English, Spanish or French, or (11) CRT trials published only in the grey literature.

### Information sources and search strategy

Literature search strategies were developed in collaboration with an academic librarian. The search strategy was limited to the period from January 2012 to May 2016 and, due to resource constraints, was not updated towards the end of the review (*12-07-2017*). Search strategies used MESH and text words related to cluster randomised trials, developing countries and public health interventions. The electronic database search was developed first for MEDLINE (ovid) and then adapted for EMBASE (ovid), CINAHL (ovid), PubMed and EMB Reviews (ovid). The full search strategy is available in an online supplement (Additional file 2). Searches were filtered to articles concerning humans, written in English, French or Spanish. We added relevant studies as suggested by the review team. Identified records were uploaded into EndNote software and duplicates were eliminated.

### Study screening and selection

Study screening and selection were performed manually within EndNote. To ensure the availability of study protocols, we limited the search to CRTs that have the word “regist*” in the abstract and used these results to begin the process of screening and selection. We validated this procedure by examining a subset of excluded articles. Screening and selection were performed in two stages by two independent reviewers (MCP and NM). In the first stage, reviewers independently screened the titles and abstracts of each identified reference against the inclusion criteria to eliminate irrelevant publications. In the second stage, each reviewer independently screened the full text of all studies that appeared to meet the inclusion criteria, or for which eligibility was uncertain. For each study, reviewers identified additional articles of potential relevance by reviewing references from the main trial report, consulting the trial registry record and searching the PubMed database for new publications by the lead trial author or using trial identifiers. To aid in article screening and selection, the team developed and tested a full text screening sheet. Any disagreements between reviewers were resolved through discussion and, as necessary, through arbitration by a third author (MJ). A summary table describing studies excluded with reasons is available in an online supplement (Additional file 3).

### Data extraction and data items

According to our protocol, two review authors would extract data independently. Given the volume and complexity of materials to review, it was not practical for both reviewers to abstract data independently. We adopted the following procedure (*20-09-2016*): included studies were randomly assigned to each of the two reviewers. Relevant data were extracted from each study protocol and trial report by one reviewer (MCP or NM) and cross-checked by the other reviewer (MCP or NM). To ensure high quality data extraction and to reach consensus, frequent meetings were held by the two reviewers (MCP and NM), including a third author (MJ) when necessary.

### Outcomes and prioritisation

The search and selection process for this review was designed to identify two quantities required for calculation of outcomes based on proportions, namely (1) numerator: these are studies that meet all inclusion and exclusion criteria. As for all systematic reviews, these studies were our principal focus and were included in the review. (2) Denominator: this is the total of studies for this review (N), which we defined as all studies that satisfy all inclusion and exclusion criteria, with the exception of the outcome criterion (planned or report IF assessment).

### Primary outcome

The primary outcome for this study was the proportion of overall agreement between the protocol and trial report concerning occurrence of IF assessment. This corresponds to research question 4. Data were summarised in a two-by-two table comparing assessment of intervention fidelity in the trial report to that in the protocol. ‘N’ represents the set of recently published CRTs of public health interventions in LMICs that have registered the study protocol in a publicly availably trial registry. For each CRT in N, we determined whether IF was assessed in the registered (or published) protocol or in the trial report (or associated documents). Studies judged to have assessed IF were coded as “1”; others were coded as “0”. The proportion of overall agreement is defined as the proportion of eligible CRTs for which the protocol and the trial report agree in the practice of intervention fidelity assessment (i.e. both positive or both negative). It was computed as (a+d) /N.

**Table:** 

	Protocol		
	+	–	
Trial Report	+ a	b	a + b
	– c	d	c + d
	a + c	b + d	N

### Secondary outcomes

To address research questions 1, 2 and 3, we also calculated:

- The frequency and proportion of trial protocols reporting assessment of intervention fidelity, out of N
- The frequency and proportion of trial reports reporting assessment of intervention fidelity, out of N
- The proportion of positive agreement among those that agree, computed as a/(a+d)
- Frequency counts and percentages summarising fidelity components examined and data collection methods proposed or employed

### Risk of bias in individual studies

We used the Cochrane Collaboration tool to assess the risk of bias in individually randomised trials [40] and included additional criteria relevant to assessing risk of bias in CRTs [40–42], which are available in an online supplement (Additional file 4). We assessed each criterion as low, high or uncertain risk, and provided a sample text that illustrates the reasons for this judgment. The initial evaluation was performed by the primary reviewer (MCP or NM) and verified by the other reviewer (MCP or NM). In case of doubt, a third reviewer (MJ) was consulted. To investigate whether study quality influences IF practices, we stratified the sample to create categories of low risk studies (7 or more of the 10 criteria were evaluated as low risk, n = 36 studies), versus uncertain or high-risk studies (6 or fewer of the 10 criteria were evaluated as low risk, n = 29 studies). Cut-points were chosen empirically to create groups of roughly equal size.

### Data synthesis

A narrative synthesis was used to explore relationships and findings within and between the included studies. As our review focuses on methodological issues, we did not assess potential publication bias.

## Results

### Study selection

**Fig. 1:**
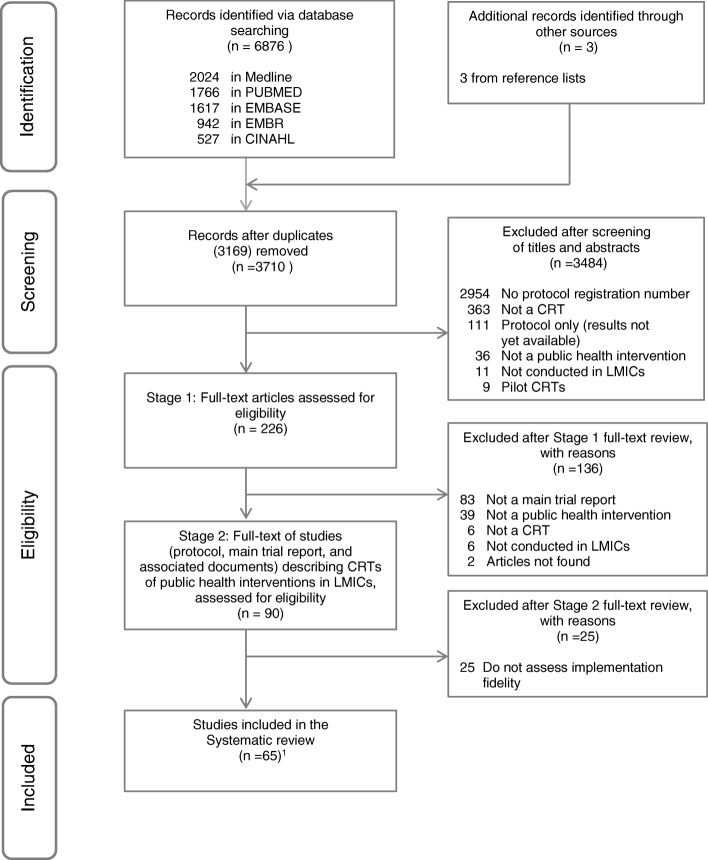
Flow diagram summarising study selection process. ^1^There are 65 studies comprising 65 protocols from trial registries, 21 published peer-reviewed protocols, 15 grey literature protocols, 65 published peer-reviewed main trial reports, 13 secondary peer-reviewed publications reporting trial results, 6 complementary grey literature documents

The electronic search identified 6876 publications, of which 226 potentially relevant studies were assessed in full text. Stage 1 of the full text review was used to define the study denominator, as follows: of the 226 articles screened, 90 were CRTs of public health interventions in LMICs with a study protocol in a publicly available trial registry published from January 2012 to May 2016. In Stage 2 of the full text review, we reviewed these 90 studies to identify those that in addition considered IF in either the protocol, the trial report or both. We excluded 25 studies (28%) that did not plan or report assessing IF. The remaining 65 studies addressed at least one IF dimension [43–107]. Figure 1 shows the PRISMA flow chart.

### Study characteristics

The 65 studies were published in English between January 2012 and May 2016. The included studies are very diverse in terms of sample size and target population, ranging from 190 to more than 280,000 eligible participants per study. Table 2 presents general characteristics of the studies included in the review. Characteristics of the individual studies included are summarised in an online supplement (Additional file 5). Interventions reviewed addressed diverse public health aims; a detailed classification is available in an online supplement (Additional file 6).

**Table 2:** General characteristics of included studies (n = 65)

Characteristics	(n)	(%)
Study designs		
Two-arm parallel cluster randomised trial (CRT)	49	75
2 × 2 factorial design CRTs	9	14
Three-arm parallel CRTs	5	8
Stepped wedge designs	2	3
Region		
Sub-Saharan Africa	36	55
South Asia	14	22
Latin America & Caribbean	6	9
East Asia & Pacific	6	9
Multiple regions	3	5
Intervention category		
HIV, tuberculosis, malaria, dengue	24	37
Child health conditions	19	29
Maternal and newborn health	14	22
Non-communicable diseases	8	12
Comparator (control group)		
Receipt of usual services or care	47	72
Applied a simplified version of the intervention	15	23
Used placebo	3	5
Intervention fidelity assessment		
Protocol		
4 dimensions	2	3
3 dimensions	5	8
2 dimensions	11	17
1 dimension	18	28
Not done	29	44
Trial		
4 dimensions	5	8
3 dimensions	18	28
2 dimensions	21	32
1 dimension	20	31
Not done	1	1

#### Outcome

We identified 90 CRTs of public health interventions in LMICs with a study protocol in a publicly available trial registry published from January 2012 to May 2016. IF assessment was planned in 40% (36/90) of trial protocols and reported in 71.1% (64/90) of trial reports. Notably, 27.8% (25/90) of studies did not assess any IF dimension in either the trial or the protocol. Practices concerning IF assessment diverged between trial protocols and reports (Table 3). The proportion of overall agreement between the trial protocol and trial report concerning occurrence of IF assessment was 66.7% (60/90). The proportion of positive agreement among those that agree was 58.3% (35/60). We were unable to compute outcomes 4b, 4c and 4d as specified in the original protocol [34].

**Table 3:** Intervention fidelity assessment among cluster randomised trials of public health interventions in low- and middle-income countries (n = 90)

		Trial Report		Total
		Positive	Negative	
Trial Protocol	Positive	35 (a)	1 (c)	36 (a + c)
	Negative	29 (b)	25 (d)	54 (b + d)
	Total	64 (a + b)	26 (c + d)	90 N (a + b + c + d)

### Practices of intervention fidelity assessment in protocols and trial reports

Of 65 studies assessing IF, 71% (46/65) described fidelity assessment only in the main trial report, 20% (13/65) of studies published a process evaluation, and 9% (6/65) of studies detailed IF in a complementary document. We found that fidelity components and moderating factors were not systematically assessed in CRTs (Table 4). In addition, only 28% (18/65) of studies monitored events taking place in control groups.

**Table 4:** Fidelity components and moderating factors identified^a^ (n = 65)

Trial Protocol			
Fidelity components	Studies		Study ID
	(n)	(%)	
Content	34	52.3	[43–76]
Coverage	13	20	[43, 44, 50, 53, 57, 62, 64, 69, 73–75, 77, 78]
Frequency	12	18.5	[43–45, 49, 50, 52, 57–59, 62, 70, 73]
Duration	4	6.2	[50, 63, 70, 73]
Trial Report			
Fidelity components	Studies		Study ID
	(n)	(%)	
Content	60	92.3	[43–60, 62–69, 71, 73–105]
Coverage	34	52.3	[43–48, 50, 52, 53, 57–59, 62–64, 66, 68, 71–75, 77, 80, 84, 85, 90, 93, 94, 97, 98, 101, 103, 105]
Frequency	33	50.8	[43–45, 48–50, 52, 57–59, 62, 63, 65, 68–70, 72–74, 78, 80–82, 84, 85, 90, 94, 99, 100, 101, 104–106]
Duration	9	13.8	[43, 47, 50, 52, 53, 68, 101, 106, 107]
Protocol & Trial			
Moderating factors	Studies		Study ID
	(n)	(%)	
Comprehensiveness of intervention description	20	30.8	[43, 48, 50, 52, 55, 59, 62, 63, 66–70, 73, 74, 79, 87, 90, 96, 106]
Strategies to facilitate implementation	60	92.3	[43–74, 76–85, 88–93, 95–102, 104–107]
Quality of delivery	22	33.8	[43–45, 52, 53, 59, 62–64, 66, 67, 70, 71, 73, 80, 85, 89, 91, 96, 100, 102, 106]
Participant responsiveness	37	56.9	[46, 48, 49, 50–53, 55, 57, 58, 62, 64–66, 69, 72–75, 78–81, 84, 87–91, 94, 96, 97, 99, 102, 104, 106, 107]
Recruitment	5	7.7	[48, 52, 73, 80, 81]
Context	8	12.3	[45, 52, 65, 66, 69, 89, 94, 106]

^a^With methods proposed or used to assess key fidelity components and moderating factors

### Methods employed to assess fidelity components and moderating factors

Among the 65 the CRTs reviewed, a variety of procedures were used to assess, maintain and monitor intervention fidelity, including (1) training in intervention delivery, (2) consultation of manuals and standardised guidelines, (3) regular meetings between personnel involved in intervention delivery and researchers to provide feedback, support and monitoring during implementation, (4) logistical and management support for trial coordinators, (5) support teams composed of local area and site managers, (6) supervision and monitoring to ensure data quality, and (7) field notes, self-reported evaluation or checklists to verify implementation progress. Researchers often reported the strategies used to promote intervention fidelity but failed to describe methods used to assess IF [108].

### Risk of bias within studies

**Fig. 2:**
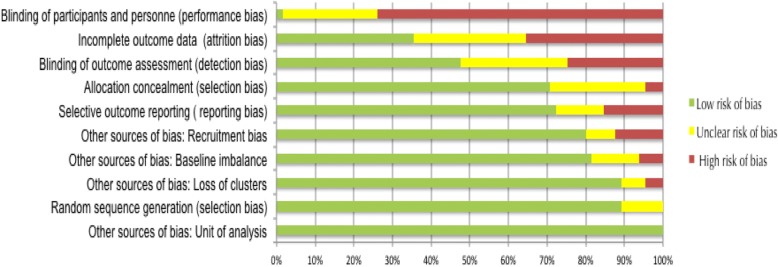
Summary of risk of bias in included randomised controlled trials

All studies reported accounting for clustering in the analysis. Due to the nature of the interventions, 74% (48/65) of the studies presented a high risk of bias in the category of ‘blinding of participants and personnel’. Only one study (1.5%) had low risk of bias for all dimensions evaluated [46]. The risk of bias graph for individual studies is available in an online supplement (Additional file 7). The overall risk of bias summary is reported in Fig. 2.

### Subgroup analyses

To better understand heterogeneity of results concerning IF assessment, we compared studies at low risk of bias versus those at uncertain or high risk of bias (combined). We found no clear differences in IF assessment between subgroups. Results are available in an online supplement (Additional file 8).

## Discussion

Fidelity assessment may contribute to making studies more reliable, internally valid and externally generalisable [109]. Our review shows that fidelity assessment is not currently a systematic practice in CRTs of public health interventions carried out in LMICs. In addition, the amount of detail given and data collection methods used for each component were variable between studies. In the absence of fidelity assessment, it may be difficult to determine if CRT results are due to the intervention design, to its implementation, or to unknown or external factors that may influence results.

### Strengths and limitations of the study

The strengths of this review include publication of the study protocol, adherence to the PRISMA guidelines, the use of five bibliographic databases providing comprehensive information on randomised trials [110], and recognising that word limits for scientific journal articles are highly constrained and that the current CONSORT reporting guidelines for CRTs do not require a description of elements related to IF, extending the search for IF assessment to associated documents beyond the main trial report.

Our study also has limitations. First, we included only CRTs that have the word “regist*” in the abstract. Therefore, we may not have identified all CRTs. However, we reviewed 16 excluded studies to assess this risk but none met our inclusion criteria. Thus, the effect of this limitation is likely to be small. Second, according to the original protocol [34], we were unable to compute outcomes for research questions 4b (Are trial reports with negative findings for the ITT analysis more likely to report a per-protocol (PP) analysis?), 4c (What is the overall agreement between ITT and PP analyses concerning intervention effectiveness?), and 4d (Does the magnitude of the intervention effect differ for PP as compared to ITT analyses?) for two main reasons. Firstly, it was not always possible to determine whether articles had performed an ITT analysis to assess the intervention effect. Although the CONSORT statement requires that authors indicate whether analysis were performed on an ITT basis, 16 (25%) studies did not explicitly declare the analysis as ITT or PP. These findings confirm that ITT analysis is “*often inadequately described and inadequately applied*” [111] in relation to missing outcome data in RCTs [111, 112]. Secondly, many articles reported more than one primary outcome; 58% (38/65) of the studies had more than one primary outcome, while 42% (27/65) of studies have results for one primary outcome. Within studies, conclusions regarding the effectiveness of interventions sometimes diverged; 55% (36/65) of studies reported at least one outcome effective.

### Strengths and weaknesses in relation to other studies

This is the first published systematic review focussing on methodological issues related to fidelity assessment in CRTs. Previous reviews have shown that the information required to ensure internal and external validity is often poorly reported in CRTs [18, 113]. This study complements previous reviews by documenting considerable variation in the practice of fidelity assessment for CRTs of public health interventions in LMICs and suggesting the potential need for methodological improvement in this area.

### Interpretation of findings

This review demonstrates that fidelity assessment is not currently a systematic practice in CRTs. Public health interventions are complex and may be affected by multiple contextual factors affecting the internal and external validity of the results. In CRTs, contextual differences at the cluster level could favour local adaptation of the intervention and differences in participants’ exposure to intervention components [114, 115]. This variability can lead to the heterogeneity of fidelity, reducing power to detect significant effects where they might be present. Fidelity assessment could help to fill this knowledge gap to aid researchers in attributing outcomes to the actual intervention and identify contextual factors that influence intervention in CRTs [18, 20, 27]. However, current reporting guidelines for CRTs offer no specific guidance on intervention fidelity assessment.

Failure to consider fidelity assessment in CRTs can be explained by several factors. First, approaches to assess intervention fidelity within randomised trials have not yet been made accessible to practitioners. Carroll et al. synthesise previous work [23, 25, 28, 109, 116], establishing a conceptual framework to evaluate intervention fidelity [116–119]. However, this framework does not provide guidance on methods to assess specific fidelity dimensions in practice. Second, fidelity assessment is not required at present by the CONSORT guidelines for CRT reporting. This does not encourage researchers to think about the importance of assessing intervention fidelity, nor to report their evaluations if conducted. Third, scientific journals limit the number of words in the final report, which may restrict authors’ ability to report their evaluations.

## Conclusions

Randomised trials are generally viewed as the gold standard for establishing evidence of intervention effectiveness. Public health interventions require high-quality evaluations to determine whether the programmes work or not and to know how to improve them [120, 121]. Results of this methodologically oriented systematic review demonstrate considerable heterogeneity in the practice of fidelity assessment in CRTs of public health interventions carried out in LMICs. Failure to assess intervention fidelity may weaken the internal and external validity of a study. We offer two recommendations to enhance the quality of future CRTs.

First, CRT reporting guidelines should promote fidelity assessment as a means to facilitate valid causal inference and replication of results. Guidelines, such as those produced by the Cochrane network and by CONSORT, play an important role in helping authors to strengthen study methods and reporting and thereby in improving the quality of health research [2, 29, 30, 40, 122, 123]. The most recent Cochrane risk of bias guidelines include, for the first time, consideration of the potential biases arising from deviations from intended interventions [122, 123]. Based on the results of this review, we make two suggestions to strengthen CRT methods and reporting. CRT guidelines such as those produced by CONSORT should promote fidelity assessment and reporting to improve the transparency of reports and to help draw firm conclusions about the outcomes of interventions. By the same principle, scientific journals should encourage researchers to conduct and report IF assessment in CRTs.

Second, a practical tool is required to guide trialists involved in the design and implementation of CRTs on how to assess IF. The TIDieR checklist encourages researchers to improve the reporting of interventions and suggests, within its 12 items, that authors describe how the fidelity of the intervention was assessed [124]. However, to our knowledge, neither the TIDieR checklist nor any other resource offers researchers a practical guide on how to approach interventionfidelity. This is a fundamental gap that should be remedied.

## Additional files

Additional file 1: PRISMA Checklist: Comparison of registered and published intervention fidelity assessment in cluster randomised trials of public health interventions in low- and middle-income countries: systematic review. (DOC 64 kb)

Additional file 2: Full search strategy for MEDLINE. (DOCX 32 kb)

Additional file 3: Articles excluded with reasons. (DOCX 38 kb)

Additional file 4: Tool for assessing risk of bias^(a)^. (DOCX 42 kb)

Additional file 5: Characteristics of included studies. (DOCX 55 kb)

Additional file 6: Classification of Public Health Interventions*. (DOCX 322 kb)

Additional file 7: Risk of bias graph for individual studies. (DOCX 136 kb)

Additional file 8: Subgroup analyses. (DOCX 19 kb)

## Abbreviations

CONSORT: Consolidated Standards of Reporting Trials
CRTs: cluster randomisation trials
IF: Intervention fidelity
ITT: intention-to-treat
LMICs: low- and middle-income countries
PP: per protocol
PRISMA: Preferred Reporting Items for Systematic Reviews and Meta-Analyses
RCTs: Randomised controlled trials
WHO: World Health Organization
