# Supplementary material for: Comparison of registered and published intervention fidelity assessment in cluster randomised trials of public health interventions in low- and middle-income countries: systematic review
Source: Trials. 2018 Jul 31;19:410. doi: 10.1186/s13063-018-2796-z (PMC6069979; doi:10.1186/s13063-018-2796-z)
Supplement: Supplementary file 3 — Articles excluded with reasons. (DOCX 38 kb) [file 13063_2018_2796_MOESM3_ESM.docx]

**Additional file 3** **Articles excluded with reasons**

- **Stage 1: Excluded after full-text review, with reasons (n=136)**

| Study | Reason for exclusion |
| --- | --- |
| Adi, A. E., et al. (2015) | Not a CRTs |
| Aliyu, M. H., et al. (2016) | Article not found |
| Allison, J. J., et al. (2016) | Not a main trial report |
| Amza, A., et al. (2012). | Not a CRTs |
| Anchala, R., et al. (2015) | Not a public health intervention |
| Anchala, R., et al. (2012). | Not a main trial report |
| Araya, R., et al. (2013). | Not conducted in LMICs |
| Arifeen, S. E., et al. (2012) | Not a public health intervention |
| Arnold, B. F., et al. (2013). | Not a main trial report |
| Aung, M. N., et al. (2012) | Not a main trial report |
| Baiden, F., et al. (2016) | Not a public health intervention |
| Banda, H. T., et al. (2015) | Not a main trial report |
| Barbosa Filho, V. C., et al. (2015) | Not a main trial report |
| Barnabas, R. V., et al. (2016) | Not a CRTs |
| Beattie, T. S., et al. (2015) | Not a main trial report |
| Bernabe-Ortiz, A., et al. (2014) | Not a main trial report |
| Berwanger, O., et al. (2012) | Not a public health intervention |
| Beune, E. J., et al. (2014) | Not conducted in LMICs |
| Birungi, N., et al. (2015) | Not a main trial report |
| Blaya, J. A., et al. (2014) | Not a public health intervention |
| Boisson, S., et al. (2014) | Not a main trial report |
| Bowen, A., et al. (2012) | Not a main trial report |
| Browning, C., et al. (2016) | Not a public health intervention |
| Cavalcanti, A. B., et al. (2016). | Not a public health intervention |
| Chan, A., et al. (2015). | Not a main trial report |
| Chandir, S., et al. (2014) | Not a CRTs |
| Chen, S., et al. (2015) | Not a public health intervention |
| Chen, Y., et al. (2014) | Not a public health intervention |
| Chen, Y., et al. (2015). | Not a main trial report |
| Chibanda, D., et al. (2015) | Not a main trial report |
| Chinbuah, M. A., et al. (2013) | Not a public health intervention |
| Christian, P., et al. (2013) | Not a main trial report |
| Coulibaly, Y. I., et al. (2013) | Not a public health intervention |
| Crea, T. M., et al. (2015). | Not a main trial report |
| Cundill, B., et al. (2015). | Not a public health intervention |
| Drummond, J., et al. (2014) | Not conducted in LMICs |
| Dryden-Peterson, S., et al. (2015). | Not a public health intervention |
| Du, X., et al. (2014) | Not a public health intervention |
| Dumont, A., et al. (2013). | Not a public health intervention |
| Durovni, B., et al. (2014). | Not a public health intervention |
| Edmond, K., et al. (2012) | Not a main trial report |
| Ellard, D., et al. (2012) | Not a main trial report |
| Engebretsen, I. M., et al. (2014) | Not a main trial report |
| Eriksson, L., et al. (2016) | Not a main trial report |
| Falb, K. L., et al. (2016) | Not a main trial report |
| Gallegos-Carrillo, K., et al. (2014) | Not a main trial report |
| Gannon, B., et al. (2014) | Not a public health intervention |
| Gibbs, L., et al. (2015) | Not conducted in LMICs |
| Halliday, K. E., et al. (2014) | Not a main trial report |
| Hargreaves, J. R., et al. (2015) | Not a main trial report |
| Henao-Restrepo, A. M., et al. (2015) | Not a main trial report |
| Hess, S. Y., et al. (2015) | Not a public health intervention |
| Hirschhorn, L. R., et al. (2015) | Not a main trial report |
| Investigators, C.-I., et al. (2015) | Not a main trial report |
| Jemmott, J. B., 3rd, et al. (2014). | Not a main trial report |
| Jenkins, R., et al. (2013) | Not a public health intervention |
| Jiamjariyaporn, T., et al. (2014) | Not a main trial report |
| Kajula, L., et al. (2016) | Not a main trial report |
| Kalyango, J. N., et al. (2013) | Not a main trial report |
| Kalyango, J. N., et al. (2013) | Not a main trial report |
| Kent, L. M., et al. (2015). | Not a main trial report |
| Kestler, E., et al. (2013) | Not a main trial report |
| Krebs, N. F., et al. (2012) | Not a public health intervention |
| Kyriacos, U., et al. (2015) | Not a public health intervention |
| Laezer, K. L., et al. (2013) | Not a main trial report |
| Lakshminarayana, R., et al. (2013). | Not a public health intervention |
| Lawal, I. U., et al. (2015). | Not a main trial report |
| Li, W. H., et al. (2015) | Not a main trial report |
| Lignou, S., et al. (2016) | Not a main trial report |
| Lin, Q., et al. (2015) | Not a main trial report |
| Lippman, S. A., et al. (2016) | Not a main trial report |
| Liu, J. Y. and C. Lai (2014) | Not a main trial report |
| Liu, X., et al. (2015) | Not a public health intervention |
| Machado, F., et al. (2015) | Not a main trial report |
| MacIntyre, C. R., et al. (2015) | Not a public health intervention |
| Martin, R. M., et al. (2013) | Not a public health intervention |
| Maselko, J., et al. (2015) | Article not found |
| Mash, B., et al. (2012) | Not a main trial report |
| Mazumder, S., et al. (2014) | Not a main trial report |
| Mbacham, W. F., et al. (2014) | Not a public health intervention |
| McClure, E. M., et al. (2014) | Not a main trial report |
| McNairy, M. L., et al. (2015) | Not a main trial report |
| Menya, D., et al. (2013) | Not a main trial report |
| Monse, B., et al. (2012) | Not a public health intervention |
| Nabulsi, M., et al. (2014) | Not a main trial report |
| Nery, S. V., et al. (2015) | Not a main trial report |
| Nsibande, D., et al. (2013) | Not a main trial report |
| Noa Noatina, B., et al. (2013) | Not a CRTs |
| Ogedegbe, G., et al. (2014) | Not a main trial report |
| Ogedegbe, G., et al. (2014) | Not conducted in LMICs |
| Ojha, S., et al. (2014) | Not a main trial report |
| Oken, E., et al. (2013). | Not a main trial report |
| Oluoch, T., et al. (2016) | Not a public health intervention |
| Operario, D., et al. (2016) | Not a public health intervention |
| Orne-Gliemann, J., et al. (2015) | Not a main trial report |
| Ota, M. O. C., et al. (2012) | Not a main trial report |
| Overgaard, H. J., et al. (2012) | Not a main trial report |
| Peltzer, K., et al. (2013) | Not a public health intervention |
| Penfold, S., et al. (2014). | Not a main trial report |
| Pettifor, A., et al. (2015) | Not a main trial report |
| Prado, E. L., et al. (2016) | Not a public health intervention |
| Prado, E. L., et al. (2012). | Not a public health intervention |
| Qu, C., et al. (2014) | Not a main trial report |
| Rabbani, F., et al. (2014) | Not a main trial report |
| Ramsey, K., et al. (2013) | Not a main trial report |
| Rasoamanamihaja, C. F., et al. (2016) | Not a CRTs |
| Robertson, L., et al. (2012) | Not a main trial report |
| Rustagi, A. S., et al. (2016) | Not a public health intervention |
| Sando, D., et al. (2014) | Not a main trial report |
| Sathish, T., et al. (2013) | Not a main trial report |
| Scott, S., et al. (2014) | Not a main trial report |
| Senarathna, L., et al. (2013) | Not a public health intervention |
| Shafique, S., et al. (2016) | Not a public health intervention |
| Shah, L., et al. (2015) | Not a main trial report |
| Shah More, N., et al. (2013) | Not a main trial report |
| Shelley, D., et al. (2015) | Not a main trial report |
| Sherr, K., et al. (2014) | Not a main trial report |
| Shet, A. S., et al. (2015) | Not a main trial report |
| Solomon, E., et al. (2014) | Not conducted in LMICs |
| Some, J. W., et al. (2015) | Not a public health intervention |
| Soofi, S., et al. (2012) | Not a public health intervention |
| Stinson, K., et al. (2014) | Not a main trial report |
| Sunguya, B. F., et al. (2013) | Not a main trial report |
| Taneja, S., et al. (2015) | Not a main trial report |
| Tine, R. C., et al. (2014) | Not a main trial report |
| Tiono, A. B., et al. (2015) | Not a main trial report |
| Tomlinson, M., et al. (2015 | Not a main trial report |
| Turan, J. M., et al. (2012) | Not a main trial report |
| Vijayakumar, L., et al. (2013) | Not a main trial report |
| Wagner, G. J., et al. (2014) | Not a main trial report |
| West, K. P., Jr., et al. (2014) | Not a public health intervention |
| Widyawati, W., et al. (2014) | Not a main trial report |
| Xu, F., et al. (2014) | Not a main trial report |
| Xu, W. H., et al. (2014) | Not a main trial report |
| Yore, J., et al. (2016) | Not a main trial report |
| Zlotkin, S., et al. (2013 | Not a public health intervention |

- **Stage 2: Excluded after full-text review of protocol, main trial report, and associated publications, with reasons (n =25)**

| Study | | Reason for exclusion |
| --- | --- | --- |
| Abdullah, F., et al. (2013). | Do not assess implementation fidelity | |
| Arrossi, S., et al. (2015). | Do not assess implementation fidelity | |
| Baird, S. J., et al. (2012) | Do not assess implementation fidelity | |
| Cunha, D. B., et al. (2013). | Do not assess implementation fidelity | |
| Deribew, A., et al. (2012). | Do not assess implementation fidelity | |
| Doherty, T., et al. (2013). | Do not assess implementation fidelity | |
| Ezeanolue, E. E., et al. (2015). | Do not assess implementation fidelity | |
| Garcia, P. J., et al. (2012). | Do not assess implementation fidelity | |
| Gebre, T., et al. (2012). | Do not assess implementation fidelity | |
| Hong, X. C., et al. (2013) | Do not assess implementation fidelity | |
| Joos, O., et al. (2016). | Do not assess implementation fidelity | |
| Kim, D. A., et al. (2015). | Do not assess implementation fidelity | |
| Labhardt, N. D., et al. (2014). | Do not assess implementation fidelity | |
| MacPherson, P., et al. (2014) | Do not assess implementation fidelity | |
| Maust, A., et al. (2015) | Do not assess implementation fidelity | |
| Miller, G., et al. (2012). | Do not assess implementation fidelity | |
| Mori, R., et al. (2015). | Do not assess implementation fidelity | |
| Pickering, A. J., et al. (2015). | Do not assess implementation fidelity | |
| Puchalski Ritchie, L. M., et al. (2015) | Do not assess implementation fidelity | |
| Qadri, F., et al. (2015). | Do not assess implementation fidelity | |
| Robertson, L., et al. (2013 | Do not assess implementation fidelity | |
| Tol, W. A., et al. (2014). | Do not assess implementation fidelity | |
| Tola, H. H., et al. (2016) | Do not assess implementation fidelity | |
| Xu, F., et al. (2015). | Do not assess implementation fidelity | |
| Yektatalab, S., et al. (2016). | Do not assess implementation fidelity | |
