# Supplementary material for: Comparison of registered and published intervention fidelity assessment in cluster randomised trials of public health interventions in low- and middle-income countries: systematic review
Source: Trials. 2018 Jul 31;19:410. doi: 10.1186/s13063-018-2796-z (PMC6069979; doi:10.1186/s13063-018-2796-z)
Supplement: Supplementary file 4 — Tool for assessing risk of bias(a). (DOCX 42 kb) [file 13063_2018_2796_MOESM4_ESM.docx]

**Additional file 4 Tool for assessing risk of bias^(a)^**

| **Domain** | **Support for judgement** |
| --- | --- |
| **Selection bias** | |
| **Random sequence generation.**  Was the allocation sequence adequately generated? | Describe the method used to generate the allocation sequence in sufficient detail to allow an assessment of whether it should produce comparable groups.   - The use of a **random component** should be sufficient for adequate sequence generation. - Sometimes restricted randomization is used to generate a sequence to ensure particular allocation ratios to the intervention groups (e.g. 1:1). Blocked randomization (random permuted blocks) is a common form of restricted randomization. Also common is stratified randomization, in which restricted randomization is performed separately within strata. |
| **Allocation concealment.**  Was the allocation sequence adequately concealed? | Describe the method used to conceal the allocation sequence in sufficient detail to determine whether intervention allocations could have been foreseen in advance of, or during, enrolment   - Defined as “the process used to ensure that the person deciding to enter a participant into a randomised controlled trial does not know the comparison group into which that individual will be allocated”   Among the different methods used to conceal allocation, **central randomization by a third party** is perhaps the most desirable. Methods using envelopes are more susceptible to manipulation than other approaches (Schulz 1995b). If investigators use envelopes, they should develop and monitor the allocation process to preserve concealment. In addition to use of sequentially numbered, opaque, sealed envelopes, they should ensure that the envelopes are opened sequentially, and only after the envelope has been irreversibly assigned to the participant. |
| **Performance bias** | |
| **Blinding of participants and personnel**  Was blinding of participants and personnel adequately addressed? | Describe all measures used, if any, to blind study participants and personnel from knowledge of which intervention a participant received. Provide any information relating to whether the intended blinding was effective.   - After enrolment into the study, **blinding** (or masking) **of study participants and personnel** may reduce the risk that knowledge of which intervention was received, rather than the intervention itself, affects outcomes. Effective blinding can also ensure that the compared groups receive a similar amount of attention, ancillary treatment and diagnostic investigations - Performance bias concerning the fidelity of the interventions, and quality of the information regarding who received what interventions, including blinding of participants and healthcare providers. |
| **Detection bias** | |
| **Blinding of outcome assessment**  Were assessors adequately blinded to what intervention a participant received**?** | Describe all measures used, if any, to blind outcome assessors from knowledge of which intervention a participant received. Provide any information relating to whether the intended blinding was effective   - Different types of people can be blinded in a clinical trial (Gøtzsche 1996, Haahr 2006):  1. participants (e.g. patients or healthy people); 2. healthcare providers (e.g. the doctors or nurses responsible for care); 3. **outcome assessors, including primary data collectors (e.g. interview staff responsible for measurement or collection of outcome data) and any secondary assessors (e.g. external outcome adjudication committees**); 4. data analysts (e.g. statisticians); and 5. manuscript writers.    The first two type of people are addressed in the tool by the item ‘Blinding of participants and personnel’. **The third is addressed by the item ‘Blinding of outcome assessment’**. The last two are not explicitly covered by the tool. |
| **Attrition bias** | |
| **Incomplete outcome data**  Data on incomplete outcomes reported? | Describe the completeness of outcome data for each main outcome, including attrition and exclusions from the analysis. State whether attrition and exclusions were reported, the numbers in each intervention group (compared with total randomized participants), reasons for attrition/exclusions where reported, and any re-inclusions in analyses performed by the review authors.   - We scored:   Overall:   - Score low risk if <10% of outcomes data is missing at the individual level AND, for trials reporting some missing data, reasons for missing data are given OR if a sensitivity analysis conducted and conclusion of bias unlikely - Score unclear risk   o   if the amount of missing outcomes data is unclear  o   if <10% outcomes data is missing but, for trials reporting some missing data, reasons for missing data are NOT given  o   if more than >10% but less than <20% of outcomes data is missing : a sensitivity analysis **^(b)^**for missing data was conducted and the results demonstrated that missing data were unlikely to bias results   - Score high risk   o   if more than >10% but less than <20% of outcomes data are missing and no sensitivity analysis conducted OR sensitivity analysis conducted & results indicate risk of bias  o   if more than >20% of outcomes data are missing  Intervention vs. control:   - Score low risk if there are no statistically significant differences in missing outcomes data between individuals in intervention and control groups p < 0.05 **^(c)^** at endline - Score unclear risk   o   if the potential for differences in missing outcomes data between individuals in intervention and control groups is not evaluated/ reported  o   if there are statistically significant differences in missing outcomes data between individuals in intervention and control groups p < 0.05 ^(c)^ addressed through imputation or sensitivity analysis   - Score high risk if there are statistically significant differences in missing outcomes data between individuals in intervention and control groups p < 0.05 ^(c)^ & these are not adequately addressed through missing values (imputation or sensitivity analysis) analysis.     « High rates of missing outcome data are present in the majority of CRTs, yet handling missing data in practice remains suboptimal. Researchers and applied statisticians should carry out appropriate missing data methods, which are valid under plausible assumptions in order to increase statistical power in trials and reduce the possibility of bias. Sensitivity analysis should be performed, with weakened assumptions regarding the missing data mechanism to explore the robustness of results reported in the primary analysis » (Fiero et all, 2016)  Scoring decision: Score high if evaluated as high risk by *either* overall or by group criteria; unclear if unclear for *both* overall and by group criteria, OR unclear in one and low in the other; low risk if low risk in both overall and by group criteria |
| **Reporting bias** | |
| **Selective reporting**  Was the study free from selective outcome reporting? | State how the possibility of selective outcome reporting was examined by the review authors, and what was found.  If the protocol is available, then outcomes in the protocol and published report can be compared. If not, then outcomes listed in the methods section of an article can be compared with those whose results are reported. Some differences between protocol and publication may be explained by legitimate changes to the protocol.   - We have analyzed the changes made in the primary outcome. If change made it was High risk. If a secondary outcome is not reported, we ignore it. A possible exception might be when the change is declared in advanced of looking at the data.   Unclear risk if the language was refined (prevalence/incidence), but the main outcome is the same |
| **Other bias*** | |
| **Recruitment bias**:  Are all eligible participants (or a random sample thereof) identified before clusters are randomised? | Recruitment bias can occur when participants are recruited to the trial after the clusters have been randomly allocated. All eligible participants ideally must be identified before clusters are randomised  « Differential individual recruitment or consent—This is when different proportions of participants are recruited to the different arms of the trial. If recruitment rates differ between groups this may lead to the risk of bias » (Puffer 2003)  CRTs can have a cohort design (same people at baseline and endline) or be based on parallel cross-sections (different groups at each time period).   - We scored high risk if randomization of clusters was done and individuals were recruited afterwards - We scored low risk if the study design was a survey pre randomization and a survey post randomization, and not the same individuals followed up. |
| **Baseline imbalance**  Was baseline comparability of clusters reported, or did the authors report statistical adjustments or randomization methods to render baseline characteristics comparable (e.g. stratified or pair-matched randomization of clusters)? | Although not a form of bias as such, the risk of baseline differences can be reduced by using stratified or pair-matched randomization of clusters.  Reporting of the baseline comparability of clusters, or statistical adjustment for baseline characteristics, can help reduce concern about the effects of baseline imbalance. |
| **Loss of clusters**  Were any clusters lost to follow up during the trial, and if so, were ad hoc missing data methods used to take this into account? | In CRTs, participants lost to follow-up can occur at the level of the cluster (cluster withdrawal or lost to follow-up, inactive cluster, merging of clusters) or the individual (participant withdrawal or lost to follow-up, or transfer from one cluster to another) and have to be omitted from the analysis this may lead to bias. Take into account any cluster / participant who withdrew or was lost to follow-up by using ad hoc missing data methods  We score :   - Low risk if less than 10% of clusters lost or if no clusters lost - Unclear risk   - if the number of clusters missing was not clear   - if more than 10% of clusters lost & [(a sensitivity analysis for missing data was conducted) & (the results demonstrated that missing data were unlikely to bias results)] - Score high risk if more than 10% of clusters lost & [(no sensitivity analysis conducted) OR (sensitivity analysis conducted & results indicate risk of bias)]   “Some trials lost complete clusters after randomisation. However, the proportion of clusters lost …was relatively low and therefore would be unlikely to introduce bias” (Puffer 2003)  « Take into account any cluster that withdrew or was lost to follow-up by using ad hoc missing data methods (+ITT) for participants included in these clusters » (Giraudeau B, Ravaud P (2009) **^(d)^** |
| **Unit of analysis**  Does the statistical analysis consider clustering, or, alternatively, is analysis conducted at the same level as the allocation? | Many cluster-randomized trials are analysed by incorrect statistical methods, not taking the clustering into account. If a cluster-randomised trial is analysed without recognition of the clustering, the analysis will ignore the possible correlation between members of the same cluster. When positively correlated observations are treated as independent, the result may be standard errors which are too small, confidence intervals which are too narrow, and P values which are too small, leading to conclusions which may be false.  One way to avoid unit-of-analysis errors in cluster-randomized trials is to conduct the analysis at the same level as the allocation, however, this might reduce the power of the study, depending on the number and size of the clusters. The ideal information to extract from a cluster-randomized trial is a direct estimate of the required effect measure (for example, an odds ratio with its confidence interval) from an analysis that properly accounts for the cluster design |

**^(a^**^)^ Synthesis from The Cochrane Handbook for Systematic Reviews of Interventions (2007).; Hayes, R. J., & Moutlon, L. H. (2009). Cluster randomised trials (Chapman & Hall/CRC Interdisciplinary Statistics) ; Donner, A., & Klar, N. (2000). Design and analysis of cluster randomization trials in health research (pp.6-10)

**^(b)^**Fiero, M. H., Huang, S., Oren, E., & Bell, M. L. (2016). Statistical analysis and handling of missing data in cluster randomized trials: a systematic review. Trials, 17(1), 72.

**^(c)^** Puffer, S., Torgerson, D., & Watson, J. (2003). Evidence for risk of bias in cluster randomised trials: review of recent trials published in three general medical journals. Bmj, 327(7418), 785-789.

**^(d)^** Giraudeau B, Ravaud P (2009) Preventing Bias in Cluster Randomised Trials. PLoS Med 6(5): e1000065. doi:10.1371/journal.pmed.1000065

**Criteria for judging risk of bias in the ‘Risk of bias’ assessment Cochrane Collaboration tool**

| RANDOM SEQUENCE GENERATION  Selection bias (biased allocation to interventions) due to inadequate generation of a randomised sequence. | |
| --- | --- |
| Criteria for a judgement of ‘Low risk’ of bias. | The investigators describe a random component in the sequence generation process such as:   - Referring to a random number table; - Using a computer random number generator; - Coin tossing; - Shuffling cards or envelopes; - Throwing dice; - Drawing of lots; - Minimization*.    *Minimization may be implemented without a random element, and this is considered to be equivalent to being random. |
| Criteria for the judgement of ‘High risk’ of bias. | The investigators describe a non-random component in the sequence generation process. Usually, the description would involve some systematic, non-random approach, for example:   - Sequence generated by odd or even date of birth; - Sequence generated by some rule based on date (or day) of admission; - Sequence generated by some rule based on hospital or clinic record number.   Other non-random approaches happen much less frequently than the systematic approaches mentioned above and tend to be obvious.  They usually involve judgement or some method of non-random categorization of participants, for example:   - Allocation by judgement of the clinician; - Allocation by preference of the participant; - Allocation based on the results of a laboratory test or a series of tests; - Allocation by availability of the intervention. |
| Criteria for the judgement of  ‘Unclear risk’ of bias. | Insufficient information about the sequence generation process to permit judgement of ‘Low risk’ or ‘High risk’. |
| ALLOCATION CONCEALMENT  Selection bias (biased allocation to interventions) due to inadequate concealment of allocations prior to assignment. | |
| Criteria for a judgement of ‘Low risk’ of bias. | Participants and investigators enrolling participants could not foresee assignment because one of the following, or an equivalent method, was used to conceal allocation:   - Central allocation (including telephone, web-based and pharmacy-controlled randomization); - Sequentially numbered drug containers of identical appearance; - Sequentially numbered, opaque, sealed envelopes. |
| Criteria for the judgement of ‘High risk’ of bias. | Participants or investigators enrolling participants could possibly foresee assignments and thus introduce selection bias, such as allocation based on:   - Using an open random allocation schedule (e.g. a list of random numbers); - Assignment envelopes were used without appropriate safeguards (e.g. if envelopes were unsealed or non­opaque or not sequentially numbered); - Alternation or rotation; - Date of birth; - Case record number; - Any other explicitly unconcealed procedure. |
| Criteria for the judgement of  ‘Unclear risk’ of bias. | Insufficient information to permit judgement of ‘Low risk’ or ‘High risk’. This is usually the case if the method of concealment is not described or not described in sufficient detail to allow a definite judgement – for example if the use of assignment envelopes is described, but it remains unclear whether envelopes were sequentially numbered, opaque and sealed. |
| BLINDING OF PARTICIPANTS AND PERSONNEL  Performance bias due to knowledge of the allocated interventions by participants and personnel during the study. | |
| Criteria for a judgement of ‘Low risk’ of bias. | Any one of the following:   - No blinding or incomplete blinding, but the review authors judge that the outcome is not likely to be influenced by lack of blinding; - Blinding of participants and key study personnel ensured, and unlikely that the blinding could have been broken. |
| Criteria for the judgement of ‘High risk’ of bias. | Any one of the following:   - No blinding or incomplete blinding, and the outcome is likely to be influenced by lack of blinding; - Blinding of key study participants and personnel attempted, but likely that the blinding could have been broken, and the outcome is likely to be influenced by lack of blinding. |
| Criteria for the judgement of  ‘Unclear risk’ of bias. | Any one of the following:   - Insufficient information to permit judgement of ‘Low risk’ or ‘High risk’; - The study did not address this outcome. |
| BLINDING OF OUTCOME ASSESSMENT  Detection bias due to knowledge of the allocated interventions by outcome assessors. | |
| Criteria for a judgement of ‘Low risk’ of bias. | Any one of the following:   - No blinding of outcome assessment, but the review authors judge that the outcome measurement is not likely to be influenced by lack of blinding; - Blinding of outcome assessment ensured, and unlikely that the blinding could have been broken. |
| Criteria for the judgement of ‘High risk’ of bias. | Any one of the following:   - No blinding of outcome assessment, and the outcome measurement is likely to be influenced by lack of blinding; - Blinding of outcome assessment, but likely that the blinding could have been broken, and the outcome measurement is likely to be influenced by lack of blinding. |
| Criteria for the judgement of  ‘Unclear risk’ of bias. | Any one of the following:   - Insufficient information to permit judgement of ‘Low risk’ or ‘High risk’; - The study did not address this outcome. |
| INCOMPLETE OUTCOME DATA  Attrition bias due to amount, nature or handling of incomplete outcome data. | |
| Criteria for a judgement of ‘Low risk’ of bias. | Any one of the following:   - No missing outcome data; - Reasons for missing outcome data unlikely to be related to true outcome (for survival data, censoring unlikely to be introducing bias); - Missing outcome data balanced in numbers across intervention groups, with similar reasons for missing data across groups; - For dichotomous outcome data, the proportion of missing outcomes compared with observed event risk not enough to have a clinically relevant impact on the intervention effect estimate; - For continuous outcome data, plausible effect size (difference in means or standardized difference in means) among missing outcomes not enough to have a clinically relevant impact on observed effect size; - Missing data have been imputed using appropriate methods. |
| Criteria for the judgement of ‘High risk’ of bias. | Any one of the following:   - Reason for missing outcome data likely to be related to true outcome, with either imbalance in numbers or reasons for missing data across intervention groups; - For dichotomous outcome data, the proportion of missing outcomes compared with observed event risk enough to induce clinically relevant bias in intervention effect estimate; - For continuous outcome data, plausible effect size (difference in means or standardized difference in means) among missing outcomes enough to induce clinically relevant bias in observed effect size; - ‘As-treated’ analysis done with substantial departure of the intervention received from that assigned at randomization; - Potentially inappropriate application of imputation. |
| Criteria for the judgement of  ‘Unclear risk’ of bias. | Any one of the following:   - Insufficient reporting of attrition/exclusions to permit judgement of ‘Low risk’ or ‘High risk’ (e.g. number randomized not stated, no reasons for missing data provided); - The study did not address this outcome. |
| SELECTIVE REPORTING  Reporting bias due to selective outcome reporting. | |
| Criteria for a judgement of ‘Low risk’ of bias. | Any of the following:   - The study protocol is available and all of the study’s pre-specified (primary and secondary) outcomes that are of interest in the review have been reported in the pre-specified way; - The study protocol is not available but it is clear that the published reports include all expected outcomes, including those that were pre-specified (convincing text of this nature may be uncommon). |
| Criteria for the judgement of ‘High risk’ of bias. | Any one of the following:   - Not all of the study’s pre-specified primary outcomes have been reported; - One or more primary outcomes is reported using measurements, analysis methods or subsets of the data (e.g. subscales) that were not pre-specified; - One or more reported primary outcomes were not pre-specified (unless clear justification for their reporting is provided, such as an unexpected adverse effect); - One or more outcomes of interest in the review are reported incompletely so that they cannot be entered in a meta-analysis; - The study report fails to include results for a key outcome that would be expected to have been reported for such a study. |
| Criteria for the judgement of  ‘Unclear risk’ of bias. | Insufficient information to permit judgement of ‘Low risk’ or ‘High risk’. It is likely that the majority of studies will fall into this category. |
| OTHER BIAS  Bias due to problems not covered elsewhere in the table. | |
| Criteria for a judgement of ‘Low risk’ of bias. | The study appears to be free of other sources of bias. |
| Criteria for the judgement of ‘High risk’ of bias. | There is at least one important risk of bias. For example, the study:   - Had a potential source of bias related to the specific study design used; or - Has been claimed to have been fraudulent; or - Had some other problem. |
| Criteria for the judgement of  ‘Unclear risk’ of bias. | There may be a risk of bias, but there is either:   - Insufficient information to assess whether an important risk of bias exists; or - Insufficient rationale or evidence that an identified problem will introduce bias. |
