# Supplementary material for: Comparison of registered and published intervention fidelity assessment in cluster randomised trials of public health interventions in low- and middle-income countries: systematic review
Source: Trials. 2018 Jul 31;19:410. doi: 10.1186/s13063-018-2796-z (PMC6069979; doi:10.1186/s13063-018-2796-z)
Supplement: Supplementary file 5 — Characteristics of included studies. (DOCX 55 kb) [file 13063_2018_2796_MOESM5_ESM.docx]

**Additional file 5 Characteristics of included studies**

| Study ID | Study Design | Country | Intervention Name | Aims | IF components | |
| --- | --- | --- | --- | --- | --- | --- |
|  |  |  |  |  | **Protocol** | **Trial reports^1^** |
| Abramsky, T., et al. (2014) | 2-arm parallel | Uganda | SASA! | To assess the community level impacts of SASA to prevent violence against women and reduce HIV-risk behaviours. | CONT  COV  FREQ | CONT  COV  FREQ  DUR |
| Althabe, F., et al. (2015) | 2-arm parallel | Argentina, Zambia, Guatemala, India, Pakistan and Kenya | ACT | To assess the feasibility, effectiveness, and safety of an intervention to increase antenatal corticosteroid use for the reduction of neonatal mortality | CONT  COV  FREQ | CONT  COV  FREQ |
| Andersson, N., et al. (2015) | 2-arm parallel | Nicaragua and Mexico | Camino Verde | To test whether community mobilization based on feedback for dengue immunological status and entomological indicators adds effectiveness to conventional dengue control | -- | CONT |
| Andrade, S., et al. (2014) | 2-arm parallel | Ecuador | ACTIVITAL | To evaluate the impact of a school-based health promotion intervention for adolescents on physical fitness indicators | -- | CONT  COV  FREQ |
| Ansah, E. K., et al. (2015) | 2-arm parallel | Ghana | -- | To examine the impact of providing rapid diagnostic tests for malaria on antimalarial dispensing, with the aim of reducing current over diagnosis and overtreatment of malaria | -- | CONT  FREQ |
| Attanasio, O. P., et al. (2014) | 2 x 2 factorial | Colombia | -- | To assess the effectiveness of an integrated early child development intervention (stimulation and micronutrient supplementation), on children's development, growth, and haemoglobin levels. | -- | CONT  FREQ |
| Aung, T., et al. (2014) | 2-arm parallel | Myanmar | -- | To test the impact of a social franchising program on uptake of oral rehydration solution plus zinc for childhood diarrhoea | COV | CONT  COV |
| Awasthi, S., et al. (2013) | 2 x 2 factorial | India | Deworming and Enhanced Vitamin A Supplementation DEVTA Project | To assess the effects of a widely practicable periodic deworming regimen (albendazole) and Vitamin A (retinol) on mortality at ages 1·0–6·0 years | CONT  FREQ | CONT  COV  FREQ |
| Ayles, H., et al. (2013) | 2 x 2 factorial | Zambia and South Africa | ZAMSTAR | To reduce the burden of tuberculosis by facilitating either rapid sputum diagnosis or integrating tuberculosis and HIV services within the community | -- | CONT |
| Baker-HenninghamH., et al. (2012) | 2-arm parallel | Jamaica | The Incredible Years Teacher Training Programme | To determine the effects of a universal pre-school-based intervention on child conduct problems and social skills at school and at home. | -- | CONT  COV  FREQ |
| Bhandari, N., et al. (2012) | 2-arm parallel | India | Training health workers to implement Integrated Management of Neonatal and Childhood Illness (IMNCI) programme | To evaluate the IMNCI programme, which integrates improved treatment of illness for children with home visits for newborn care, to inform its scale-up | -- | CONT  COV  FREQ |
| Bird, C., et al. (2014) | 2-arm parallel | Zanzibar | -- | To test whether footwear reduces prevalence and intensity of hookworm infection in school-aged children | -- | CONT |
| Boisson, S., et al. (2013) | 2-arm parallel | India | -- | To evaluate the effect of promotion and free distribution of sodium dichloroisocyanurate (NaDCC) disinfection tablets for household water treatment in preventing diarrhoea prevalence among children under five. | CONT | CONT  COV |
| Boone, P., et al. (2016) | 2-arm parallel | Guinea-Bissau | The EPICS trial: Enabling Parents to Increase Child Survival | To assess whether a community-based intervention package in the absence of health system strengthening activities could generate a rapid and cost-effective reduction in under-5 mortality in these regions. | CONT | CONT  COV  DUR |
| Bousema, T., et al. (2016) | 2-arm parallel | Kenya | -- | To measure the effect of hotspot-targeted interventions with larviciding, distribution of long-lasting insecticide-treated nets, indoor residual spraying, and focal mass drug administration in evaluation zones surrounding malaria hotspots. | CONT | CONT  COV  FREQ |
| Burnhams, N. H., et al. (2015) | 2-arm parallel | South Africa | -- | To test the effectiveness of Team Awareness (TA), a programme aimed at reducing the risky use of alcohol and alcohol-related HIV risk and increasing help-seeking behaviour among a sample of employees. | -- | CONT |
| Chen-Hussey, V. et al.  (2013) | 2-arm parallel | Lao PDR | -- | To determine whether using a topical 15% DEET repellent, would reduce malaria incidence against exophagic vectors amongst rural populations using LLINs. | CONT  FREQ | CONT  FREQ |
| Clasen, T., et al. (2014) | 2-arm parallel | India | -- | To assess the effectiveness of a rural household sanitation intervention (provision of household latrines) to prevent diarrhoea, soil-transmitted helminth infection, and child malnutrition. | CONT  COV  FREQ  DUR | CONT  COV  FREQ  DUR |
| Coates, T. J., et al. (2014) | 2-arm parallel | South Africa, Tanzania, Zimbabwe and Thailand | -- | To investigate whether a social and behavioural prevention strategy could reduce HIV incidence, increase HIV testing, reduce HIV risk behaviour, and change social and behavioural norms. | CONT | CONT |
| Colbourn, T., et al. (2013) | 2 x 2 factorial | Malawi | -- | To investigate whether (1) a rural participatory women’s group community intervention, and (2) a quality improvement intervention at health centres, are effective in reducing deaths in three districts of Malawi. | CONT  FREQ | CONT  COV  FREQ  DUR |
| Corbel, V., et al. (2012) | 2 x 2 factorial | Benin | -- | To investigate whether the combination of long-lasting insecticidal mosquito nets (LLINs) with indoor residual spraying (IRS) or carbamate-treated plastic sheeting (CTPS) conferred enhanced protection against malaria and better management of pyrethroid-resistance in vectors than did LLINs alone | CONT  COV | CONT  COV  DUR |
| Dave, P. V., et al. (2016) | 2-arm parallel | India | -- | Non-inferiority trial to assess the effect of using family members as DOT providers on treatment success rates among children with newly diagnosed TB. | -- | CONT |
| Deressa, W., et al. (2014). | 2-arm parallel | Ethiopia | -- | To determine the effect of combining community-based mosquito repellent with LLINs in the reduction of malaria | CONT | CONT |
| Devries, K. M., et al. (2015) | 2-arm parallel | Uganda | The Good Schools Study | To determine whether the Good School Toolkit could reduce physical violence from school staff to students | CONT | CONT |
| Durovni, B., et al. (2013) | step wedge | Brazil | -- | To assess the effect of widespread use of isoniazid preventive therapy on rates of tuberculosis and death in people with HIV in Brazil. |  | CONT |
| Fairall, L., et al. (2012) | 2-arm parallel | South Africa | Streamlining Tasks and Roles to Expand Treatment and Care for HIV (STRETCH) programme | To assess the effects on mortality, viral suppression, and other health outcomes and quality indicators of the STRETCH programme, which provides educational outreach training of nurses to initiate and represcribe ART, and to decentralise care. | -- | FREQ  DUR |
| Feikin, D. R., et al. (2014) | 2-arm parallel | Kenya | -- | To investigate whether providing zinc at home for future diarrhoea treatment had an impact at the community level on the reduction of diarrhoea incidence, reduction of respiratory disease incidence, and increased diarrhoea episodes treated with zinc, as compared to having zinc available in the clinic only | CONT | CONT |
| Fottrell, E., et al. (2013) | 2-arm parallel | Bangladesh | -- | To assess the effect of a participatory women’s group intervention involving participatory learning and action cycles with higher population coverage on neonatal mortality in Bangladesh | -- | CONT  COV  FREQ |
| Fylkesnes, K., et al. (2013) | 2-arm parallel | Zambia | -- | To assess the impact of a home-based voluntary human immunodeficiency virus (HIV) counselling and testing model on acceptance, equity in uptake, and negative life events, compared to standard testing services. | -- | CONT |
| Go, V. F., et al. (2015) | 2 x 2 factorial | China | -- | To develop and evaluate a behavioural intervention for HIV-infected PWID involving (1) individual-level post test counselling and skill-building support groups, (2) a structural-level community stigma reduction program, (3) both individual and structural level activities, as compared to (4) standard of care HIV testing and counselling. | CONT  COV  FREQ | CONT  COV  FREQ |
| Gong, J., et al. (2015) | 2-arm parallel | China | Keep Moving toward Healthy Heart and Healthy Brain (KM2H2) | To evaluate the efficacy of the KM2H2 program in encouraging physical activities for the prevention of heart attack and stroke among hypertensive patients enrolled in the Community-Based Hypertension Control Program (CBHCP). | -- | CONT |
| Gunawardena, N., et al. (2016) | 2-arm parallel | Sri Lanka | -- | To examine the effect of a school-based intervention to enable school children to act as change agents on weight, physical activity and diet of their mothers | -- | DUR |
| Halliday, K. E., et al. (2014) | 2 x 2 factorial | Kenya | -- | To investigate the effect of (1) intermittent screening and treatment (IST) for malaria alone; (2) a literacy intervention alone; (3) both interventions combined; or  (4) control group where neither intervention was implemented, on the health and education of school children in an area of low to moderate malaria transmission. | CONT  FREQ | CONT  COV  FREQ |
| Hanson, C., et al. (2015) | 2-arm parallel | Tanzania | -- | To develop, implement and evaluate the effectiveness and cost of interventions at community level (focussed on a community-based health worker) and of health system strengthening on newborn survival in rural southern Tanzania | CONT  FREQ | CONT  COV  FREQ |
| He, F. J., et al. (2015) | 2-arm parallel | China | School-EduSalt | To determine whether an education programme targeted at schoolchildren can lower salt intake in children and their families. | CONT | CONT |
| Huybregts, L., et al. (2012) | 2-arm parallel | Chad | Prevent Acute Child Malnutrition (PREAMA) | To examine the effect of a ready-to-use supplementary food (RUSF) on prevention of wasting in 6- to 36-mo-old children within the context of a general food distribution program | CONT | -- |
| Jack, S. J., et al. (2012) | 2-arm parallel | Cambodian | -- | To evaluate the effectiveness of micronutrient Sprinkles alongside infant and young child feeding (IYCF) education compared with IYCF education alone on anaemia, deficiencies in iron, vitamin A, and zinc, and infant growth. | -- | CONT  COV |
| Kirkwood, B. R., et al. (2013) | 2-arm parallel | Ghana | Newborn home visits neonatal mortality trial (NEWHINTS) | To assess the effect of home visits by community-based surveillance volunteers to pregnant women and their babies on all-cause neonatal mortality rate and essential newborn-care practices. | CONT  COV  FREQ | CONT  COV  FREQ |
| Lewycka, S., et al. (2013) | 2 x 2 factorial | Malawi | MaiMwana | To assess whether community mobilisation through women’s groups, and health education through female volunteer peer counsellors affect rates of infant care, feeding, morbidity, and mortality | CONT  DUR | CONT  COV  FREQ |
| Lutge, E., et al. (2013) | 2-arm parallel | South Africa | -- | To test the feasibility and effectiveness of delivering economic support (vouchers) to improve pulmonary tuberculosis treatment outcomes | -- | CONT  COV  FREQ |
| Mengistie, B., et al. (2013) | 2-arm parallel | Ethiopia | -- | To assess the effectiveness of household water chlorination in reducing incidence of diarrhoea among children <5 years of age. | -- | CONT |
| More, N. S., et al. (2012) | 2-arm parallel | Mumbai | -- | To test whether slum-dweller women’s groups discussing perinatal health, lead to improved knowledge through peer learning, and development and implementation of local strategies to improve perinatal care and outcomes. | CONT  COV | CONT  COV |
| Nikiema, L., et al. (2014) | 3-arm parallel | Burkina Faso | LUCOMA | To compare the effectiveness of weekly child-centred counselling (CCC), with an improved cornsoy blend [corn-soy blend with added micronutrients (CSB++)] or a locally produced ready-to-use supplementary food (RUSF), in treating moderate acute malnutrition through first-line rural health services. | CONT | CONT  FREQ |
| Olney, D. K., et al. (2015) | 3-arm parallel | Burkina Faso | EHFP (enhanced-homestead food production program) | To assess the impact of EHFP, Helen Keller International’s integrated agriculture and nutrition and health behaviour change communication program. | -- | CONT |
| Ononge, S., et al. (2015) | step wedge | Uganda | -- | To determine whether antenatal distribution of misoprostol for pregnant women to self-administer during home births reduces postpartum haemorrhage. | -- | CONT  COV |
| Onwujekwe, O., et al. (2015) | 3-arm parallel | Nigeria | -- | To evaluate the effectiveness of provider training alone or in combination with a school-based community intervention in improving adherence to malaria treatment guidelines | CONT | CONT  COV |
| Pasha, O., et al. (2013) | 2-arm parallel | India Pakistan, Kenya, Zambia, Guatemala and Argentina | EmONC | To evaluate the impact of a comprehensive intervention of community mobilization, birth attendant training and improvement of quality of care in health facilities on perinatal mortality | CONT | CONT |
| Patil, S. R., et al. (2014) | 2-arm parallel | India | Total Sanitation Campaign (TSC) | To measure the effect of the TSC implemented with capacity building support from the World Bank’s Water and Sanitation Program on availability of individual household latrines, defecation behaviours, and child health. | -- | CONT  COV |
| Pinder, M., et al. (2015) | 2-arm parallel | Gambian | -- | To assess whether the addition of DDT indoor residual spraying to long-lasting insecticide-treated nets provides a significantly different level of protection against clinical malaria in children or against house entry by vector mosquitoes. | CONT | CONT  COV  FREQ  DUR |
| Raj, A., et al. (2016) | 2-arm parallel | India | CHARM | To evaluate the impact of a gender-equity focused, male-centered family planning intervention on marital contraceptive use and incident pregnancy. | CONT  COV | CONT  FREQ |
| Rotheram-Borus, M. J., et al. (2014) | 2-arm parallel | South Africa | Project Masihambisane | To evaluate the effect of clinic-based support by HIV-positive Peer Mentors, in addition to standard clinic care, on maternal and infant well being among Women Living with HIV. | CONT  FREQ  DUR | FREQ |
| Sangoro, O., et al. (2014) | 2-arm parallel | Tanzania | -- | To assess whether 15% DEET topical repellent in combination with long-lasting insecticide-treated nets (LLINs) can prevent greater malaria transmission, compared with placebo and LLINs | -- | CONT  FREQ |
| Singla, D. R., et al. (2015) | 2-arm parallel | Uganda | -- | To assess the effects of an integrated, community-based parenting intervention based on social-cognitive learning theory that targeted child development and maternal wellbeing | -- | CONT  FREQ |
| Soofi, S., et al. (2012) | 2-arm parallel | Pakistan | -- | To establish whether community case identification and management of severe pneumonia by oral amoxicillin delivered through community health workers has the potential to reduce the number of infants dying at home. | CONT | CONT  COV |
| Sorensen, G., et al. (2016) | 2-arm parallel | India | Mumbai Worksite Tobacco Control Study | To test the effectiveness of a worksite in increasing tobacco use cessation among workers in the manufacturing sector. | -- | CONT  COV  FREQ  DUR |
| Suchdev, P. S., et al. (2012) | 2-arm parallel | Kenya | -- | To evaluate the effect of community based marketing and distribution of Sprinkles micronutrient powder (MNP) on childhood rates of anaemia and iron and vitamin A deficiency. | COV | CONT  FREQ |
| Tian, M., et al. (2015) | 2-arm parallel | China and India | SimCard | To develop and evaluate a simplified cardiovascular management program delivered by community health workers with the aid of a smartphone-based electronic decision support system on patient-reported antihypertensive medication use. | CONT | COV  FREQ |
| Tripathy, P., et al. (2016) | 2-arm parallel | India | JOHAR | To assess the impact of a community mobilization intervention with women’s groups facilitated by Accredited Social Health Activists (ASHAs) to improve maternal and newborn health outcomes | CONT  COV  FREQ  DUR | CONT  COV  FREQ |
| Waiswa, P., et al. (2015) | 2-arm parallel | Uganda | Uganda Newborn Survival Study | To assess the effect of a community health worker home visit strategy combined with health facility strengthening on uptake of newborn care seeking, practices and services, and to link the results to national policy and scale-up. | CONT  COV | CONT  COV  FREQ |
| Wang, P. C., et al. (2015) | 3-arm parallel | Southern Zambia | -- | To assesse the integration of early infant HIV diagnosis with the expanded programme for immunization to determine whether infant and postpartum maternal HIV testing rates increase without harming immunization uptake. | CONT  COV | CONT  COV |
| Weiss, S. M., et al. (2015) | 2-arm parallel | Zambia | Spear and Shield Intervention | To test the effect of a community-level intervention designed to increase demand for voluntary medical male circumcision among hard-to-reach men. | -- | CONT |
| West, P. A., et al. (2014) | 2-arm parallel | Tanzania | -- | To investigate whether the combination of indoor residual spraying with bendiocarb and long lasting Insecticide treated mosquito nets (ITNs) provides added protection compared to ITNs alone. | -- | CONT  COV |
| Yotebieng, M., et al. (2015) | 3-arm parallel | DR Congo | -- | To assess the effect of a short-cut implementation of the Ten Steps to Successful Breastfeeding programme, a key component of the Baby- Friendly Hospital Initiative. | CONT | CONT |
| Young, S. D., et al. (2015) | 2-arm parallel | Peru | Harnessing Online Peer Education (HOPE) | To examine the efficacy of the HOPE social media intervention to increase HIV testing among men who have sex with men (MSM). | -- | CONT  FREQ |
| Yousafzai, A. K., et al. (2014) | 2 x 2 factorial | Pakistan | -- | To investigate the feasibility and effectiveness of the integration of interventions to enhance child development and growth outcomes in the Lady Health Worker (LHW) programme. | -- | CONT  COV  FREQ |

^1^ Trial reports include the main study report, and relevant results from peer-reviewed and grey literature if applicable

Abbreviations: CONT – Content, COV –Coverage, FREQ – Frequency, DUR – Duration
