# Supplementary material for: Comparison of registered and published intervention fidelity assessment in cluster randomised trials of public health interventions in low- and middle-income countries: systematic review
Source: Trials. 2018 Jul 31;19:410. doi: 10.1186/s13063-018-2796-z (PMC6069979; doi:10.1186/s13063-018-2796-z)
Supplement: Supplementary file 6 — Classification of Public Health Interventions*. (DOCX 322 kb) [file 13063_2018_2796_MOESM6_ESM.docx]

**Additional file 6 Classification of Public Health Interventions***


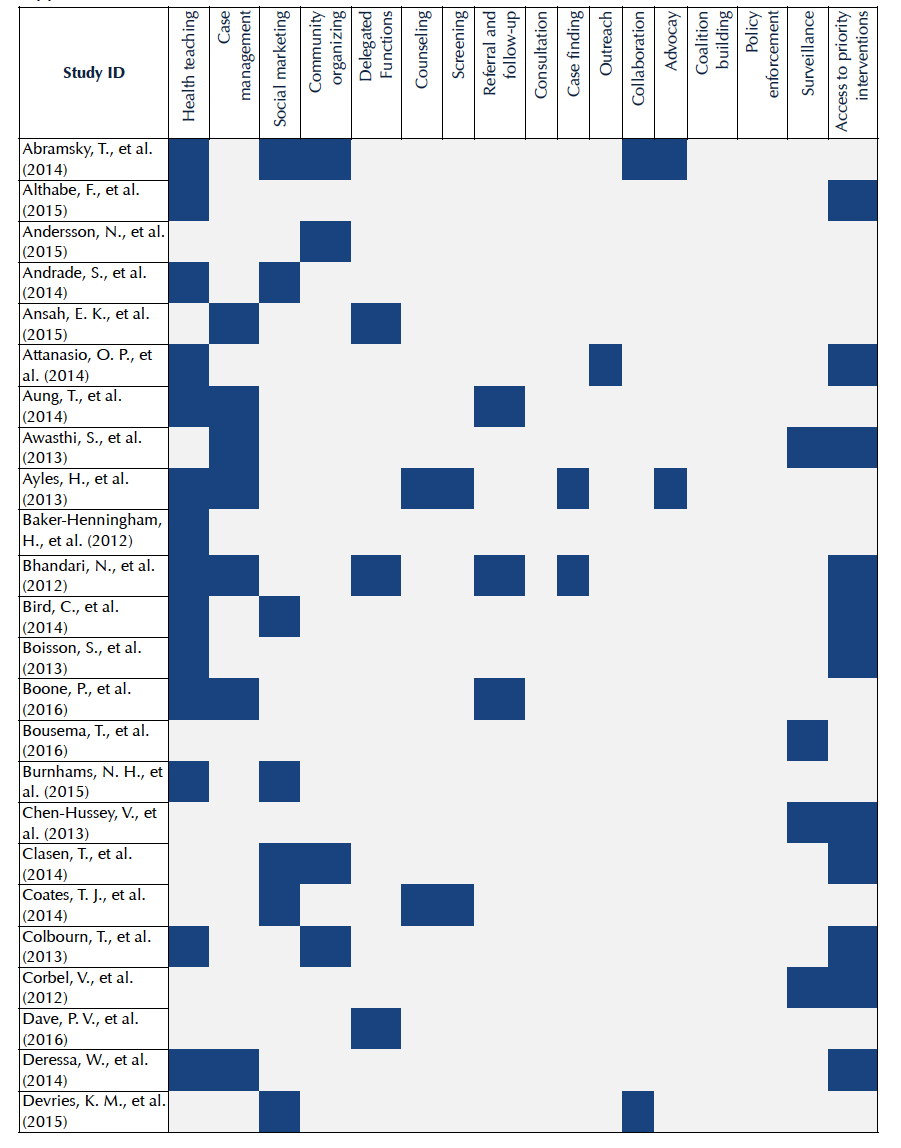


**
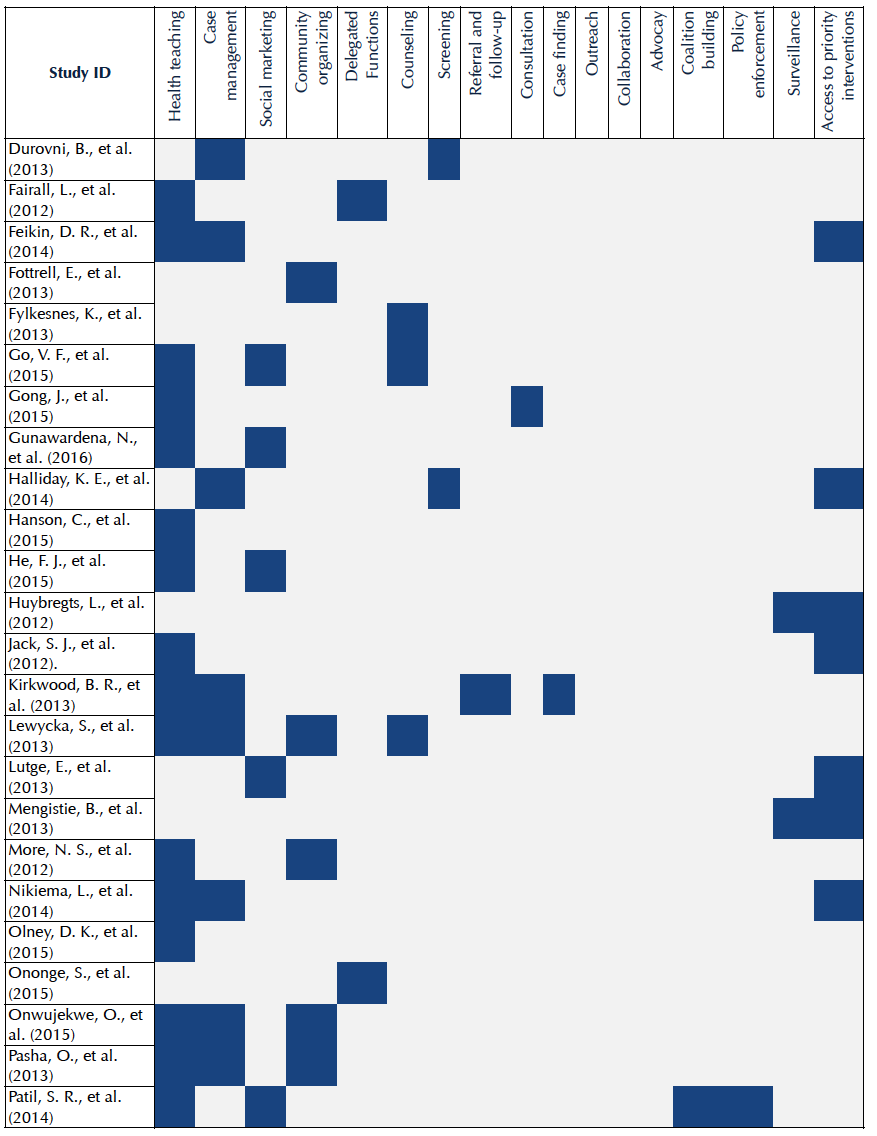
**

**
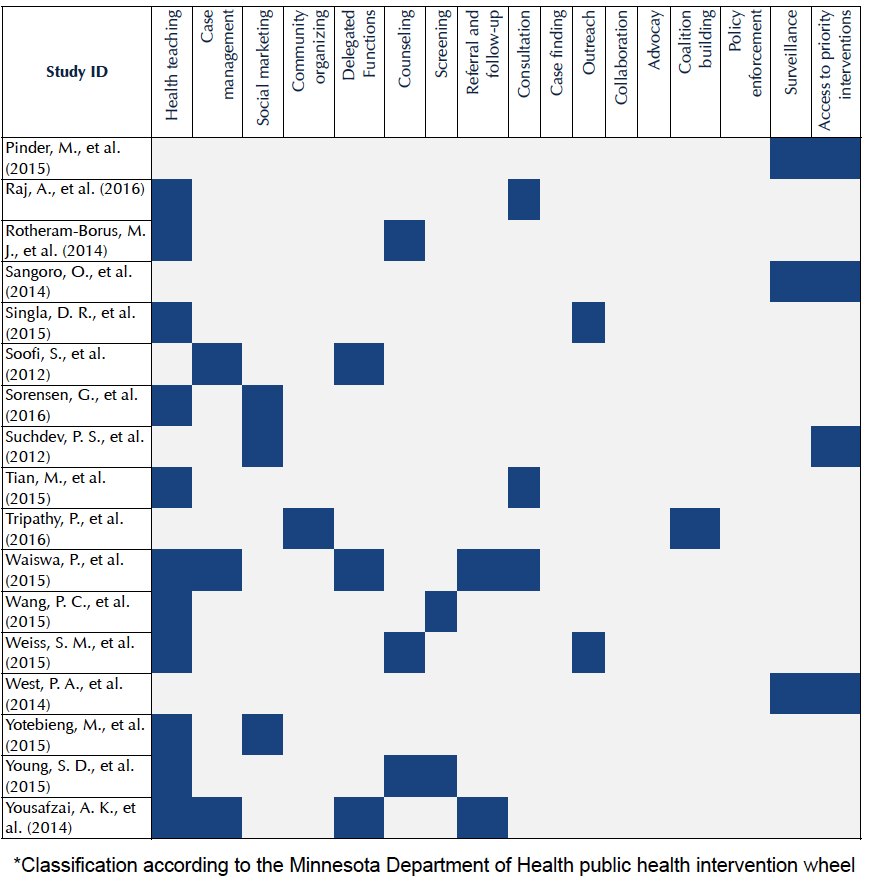
**

We included an additional category “Access to priority interventions” to represent public health initiatives seeking to increase access to key heath services and commodities
