# Supplementary figures and images for: Comparison of registered and published intervention fidelity assessment in cluster randomised trials of public health interventions in low- and middle-income countries: systematic review
Source: Trials. 2018 Jul 31;19:410. doi: 10.1186/s13063-018-2796-z (PMC6069979; doi:10.1186/s13063-018-2796-z)

**Additional file 7 Risk of bias graph for individual studies**


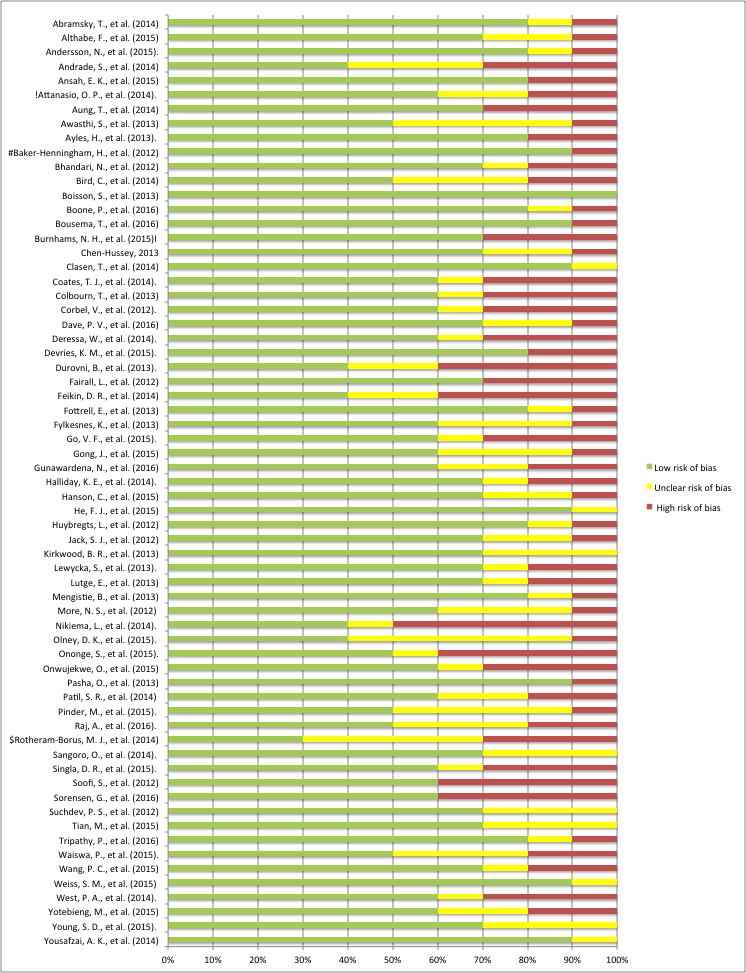

Supplement: Supplementary file 7 — Risk of bias graph for individual studies. (DOCX 136 kb) [file 13063_2018_2796_MOESM7_ESM.docx]
