# Supplementary material for: Comparison of registered and published intervention fidelity assessment in cluster randomised trials of public health interventions in low- and middle-income countries: systematic review
Source: Trials. 2018 Jul 31;19:410. doi: 10.1186/s13063-018-2796-z (PMC6069979; doi:10.1186/s13063-018-2796-z)
Supplement: Supplementary file 8 — Subgroup analyses. (DOCX 19 kb) [file 13063_2018_2796_MOESM8_ESM.docx]

**Additional file 8 Subgroup analyses**

**a. Registered and published intervention fidelity assessment, by risk of bias category (N=65)**

| Low risk of bias category^1^ (n=36) | | | | |
| --- | --- | --- | --- | --- |
|  | | **IF Assessment*** | | **Study ID** |
|  | | (n) | (%) |  |
| Protocol only | | 1 | 2.8 | 61 |
| Trial report only | | 16 | 44.4 | 78,80,83-85,87,88,90,93-95,99,102,104-106 |
| Both protocol and trial report | | 19 | 52.8 | 43,44,46-50,55,58-60,62,63,67,72,73,75,77,82 |
| Uncertain or high risk of bias category^2^ (n=29) | | | | |
|  | | **IF Assessment*** | | **Study ID** |
|  | (n) | | (%) |  |
| Protocol only | | 0 | 0 |  |
| Trial report only | | 13 | 44.8 | 79,81,86,89,91,92,96-98,100,101,103,107 |
| Both protocol and trial report | | 16 | 55.2 | 45,51-54,56,57,64-66,68-71,74,76 |

*IF assessment was judged to be present if an article proposed or used methods to assess 1 or more key fidelity components

^1.^ Low Risk = 7 to 10 our of 10 criteria scored as low risk of bias n=36

^2.^ Uncertain or High Risk = 0 -6 out of 10 criteria scored as low risk of bias n= 28

**b. Comprehensiveness of registered and published intervention fidelity assessment*, by risk of bias category**

| Low risk of bias category^1^ (n=36) | | | | | | |
| --- | --- | --- | --- | --- | --- | --- |
| **Fidelity components** | **IF planned**  **(Protocol)** | | **Study ID** | **IF performed**  **(Trial Report)** | | **Study ID** |
|  | (n) | (%) |  | (n) | (%) |  |
| 4 dimensions | 2 | 5.6 | 50,73 | 2 | 5.6 | 43,50 |
| 3 dimensions | 3 | 8.3 | 43,44, 62 | 13 | 36.1 | 44,47,48,58,59,62,63,73,84,85, 90,94,105 |
| 2 dimensions | 5 | 13.9 | 49,58,59,63,75 | 11 | 30.6 | 46,49,72,75,77,80,82,93,99,104,106 |
| 1 dimension | 10 | 27.8 | 46,47,48,55,60,61,67, 72,77,82, | 9 | 25.0 | 55,60,67,78,83,87,88,95,102 |
| Not done | 16 | 44.4 | 78,80,83-85,87,88,90, 93-95,99, 102, 104-106 | 1 | 2.8 | 61 |
| Uncertain or high risk of bias category^2^ (n=29) | | | | | | |
| **Fidelity components** | **IF planned**  **(Protocol)** | | **Study ID** | **IF performed**  **(Trial Report)** | | **Study ID** |
|  | (n) | (%) |  | (n) | (%) |  |
| 4 dimensions | 0 | 0 | - | 3 | 10.3 | 52,68,101 |
| 3 dimensions | 2 | 6.9 | 57,70 | 5 | 17.2 | 45,53,57,74,79 |
| 2 dimensions | 6 | 20.7 | 45,52,53,64,69,74 | 10 | 34.5 | 64-66,69,71,81,97,98,100,103 |
| 1 dimension | 8 | 27.6 | 51,54,56,65,66,68,71, 89 | 11 | 37.9 | 51,54,56,70,76, 86, 89, 91, 92, 96,107 |
| Not done | 13 | 44.8 | 76,79,81,86,91,92,96, 97, 98,100,101,103,107 | 0 | 0 | - |

*IF assessment was judged to be present if an article proposed or used methods to assess 1 or more key fidelity components

^1.^ Low Risk = 7 to 10 our of 10 criteria scored as low risk of bias n=36

^2.^ Uncertain or High Risk = 0 -6 out of 10 criteria scored as low risk of bias n= 28
